# Novel conditionally replicating adenovirus-mediated efficient detection of circulating tumor cells in lung cancer patients

**DOI:** 10.1371/journal.pone.0286323

**Published:** 2023-10-19

**Authors:** Sena Ikemoto, Fuminori Sakurai, Sora Tokuoka, Tomoki Yamashita, Kosuke Takayama, Kazuaki Hoshi, Takahiro Okabe, Issei Sumiyoshi, Shinsaku Togo, Kazuhisa Takahashi, Masashi Tachibana, Hiroyuki Mizuguchi

**Affiliations:** 1 Laboratory of Biochemistry and Molecular Biology, Graduate School of Pharmaceutical Sciences, Osaka University, Osaka, Japan; 2 Department of Minimally Invasive Next-generation Cancer Diagnosis by TelomeScan, Tokyo, Japan; 3 Leading Center for the Development and Research of Cancer Medicine, Juntendo University, Tokyo, Japan; 4 Division of Respiratory Medicine, Juntendo University Faculty of Medicine & Graduate School of Medicine, Tokyo, Japan; 5 The Center for Advanced Medical Engineering and Informatics, Osaka University, Osaka, Japan; 6 Center for Infectious Disease Education and Research (CiDER), Osaka University, Osaka, Japan; 7 Laboratory of Functional Organoid for Drug Discovery, Center for Drug Discovery Resources Research, National Institutes of Biomedical Innovation, Health and Nutrition, Osaka, Japan; 8 Integrated Frontier Research for Medical Science Division, Institute for Open and Transdisciplinary Research Initiatives (OTRI), Osaka University, Osaka, Japan; Universitat des Saarlandes, GERMANY

## Abstract

Circulating tumor cells (CTCs) are present in the blood of cancer patients from the early stage of cancer development, and their presence has been correlated with patient prognosis and treatment responses. Accordingly, CTCs have been attracting attention as a novel biomarker for early detection of cancer and monitoring of treatment responses. However, since patients typically have only a few CTCs per milliliter of blood, development of an accurate and highly sensitive CTC detection method is crucial. We previously developed a CTC detection method using a novel conditionally replicating adenovirus (Ad) that expresses green fluorescence protein (GFP) in a tumor cell-specific manner by expressing the E1 gene using a tumor-specific human telomerase reverse transcriptase (hTERT) promoter (rAdF35-142T-GFP). CTCs were efficiently detected using rAdF35-142T-GFP, but GFP expression levels in the CTCs and production efficiencies of rAdF35-142T-GFP were relatively low. In this study, in order to overcome these problems, we developed four types of novel GFP-expressing conditionally replicating Ads and examined their ability to visualize CTCs in the blood samples of lung cancer patients. Among the four types of novel recombinant Ads, the novel conditionally replicating Ad containing the 2A peptide and the GFP gene downstream of the E1A gene and the adenovirus death protein (ADP) gene in the E3 region (rAdF35-E1-2A-GFP-ADP) mediated the highest number of GFP-positive cells in the human cultured tumor cell lines. Titers of rAdF35-E1-2A-GFP-ADP were significantly higher (about 4-fold) than those of rAdF35-142T-GFP. rAdF35-E1-2A-GFP-ADP and rAdF35-142T-GFP efficiently detected CTCs in the blood of lung cancer patients at similar levels. GFP+/CD45- cells (CTCs) were found in 10 of 17 patients (58.8%) for both types of recombinant Ads.

## Introduction

Circulating tumor cells (CTCs), which are tumor cells circulating in the peripheral blood, have been attracting attention as a new cancer biomarker. Previous studies reported that CTCs were present in the blood of cancer patients from the early stage of cancer development and that the numbers of CTCs were correlated with patient prognosis and treatment efficacies [1–4]. In addition, because CTCs are living cancer cells, their detection provides definitive proof of the presence of tumors. The CellSearch^TM^ System has been approved by the U.S. Food and Drug Administration (FDA) as a CTC detection system. In this system, CTCs are detected as epithelial cell adhesion molecule (EpCAM)/cytokeratin-positive cells in the blood; however, it is difficult to detect CTCs undergoing epithelial-mesenchymal transition (EMT) by the CellSearch^TM^ System, because EpCAM expression declines on tumor cells undergoing EMT [5–7]. It has become clear that EMT-induced CTCs (EMT-CTCs) have increased metastatic potential [8] and that EMT-CTCs are a biomarker of patient prognosis and chemotherapy resistance [1]. Detection of EMT-CTCs is thus important to predict patient prognosis and the efficacy of cancer treatment. In order to efficiently detect EMT-CTCs, several novel CTC detection methods based on differences in the physical properties between CTCs and blood cells, including cell size, cellular surface charge, and density, have been developed [9–11]. However, these methods are often inefficient and lacking in specificity, although these methods are relatively easy to operate and cost-effective.

In order to address the above-mentioned problem of a reduction in EpCAM expression on tumor cells undergoing EMT, a CTC detection system using a green fluorescence protein (GFP)-expressing conditionally replicating adenovirus (Ad) has been developed [1, 6, 12, 13]. The conditionally replicating Ads contained the tumor-specific promoter-driven E1 gene expression cassette and the cytomegalovirus (CMV) promoter-driven GFP expression cassette. The conditionally replicating Ads showed tumor cell-specific replication, resulting in tumor cell-specific GFP expression. CTCs were detected as GFP-positive cells. Furthermore, we added the following modifications to the conditionally replicating Ads in order to detect CTCs more efficiently [1, 6]. First, the fiber protein was substituted with that of Ad serotype 35 (Ad35), which recognizes human CD46 as an infection receptor [14]. The conventional conditionally replicating Ad was based on species C Ad serotype 5, which recognizes coxsackievirus and adenovirus receptor (CAR). CAR expression is often downregulated on malignant tumor cells and tumor cells undergoing EMT [6, 15–17], whereas CD46 is ubiquitously expressed on almost all cells except for erythrocytes. In addition, CD46 expression is up-regulated on malignant tumor cells [18, 19]. Substitution with the fiber protein of Ad35 leads to efficient detection of CAR-negative CTCs. Second, miR-142T-3p-targeted sequences were inserted into the 3’-untranslated region (UTR) of the E1 gene and GFP gene. miR-142T-3p is expressed in a blood cell-specific manner [6, 20], resulting in significant suppression of GFP expression in normal blood cells. The conditionally replicating Ad containing the modifications described above, rAdF35-142T-GFP, efficiently detected the CTCs in the blood samples of lung cancer patients without significant production of false-positive cells [6] however, higher levels of GFP expression are preferable for efficient detection of CTCs.

In this study, we have developed four types of novel conditionally replicating Ads expressing GFP and examined their CTC detection efficiencies in the blood samples of lung cancer patients. Among the four types of recombinant Ads, rAdF35-E1-2A-GFP-ADP, which contained the E1A gene fused with the 2A peptide-coding sequence, the GFP gene in the E1 region and the ADP gene in the E3 region, mediated the highest GFP expression in the human cultured tumor cells, and detected CTCs in the blood samples at levels comparable to rAdF35-142T-GFP developed in the previous study [6].

## Materials and methods

### Cell lines

H1299 (a human non-small cell lung cancer cell line), MCF-7 (a human breast cancer cell line), THP-1 (a human monocyte-derived cell line), and human peripheral blood mononuclear cells (PBMCs) (Precision for Medicine, Frederick, MD) were cultured in RPMI 1640 containing 10% fetal bovine serum (FBS), 100U/mL penicillin, and 100μg/mL streptomycin. HepG2 (a human liver carcinoma-derived cell line, JCRB1054, JCRB Cell Bank, Tokyo, Japan), T24 (a human bladder carcinoma-derived cell line), and PANC-1 cells (a human pancreatic carcinoma-derived cell line) were grown in Dulbecco’s modified eagle medium (DMEM) containing 10% FBS, 100U/mL penicillin, and 100μg/mL streptomycin. All cells were cultured at 37°C in a humidified atmosphere of 5% CO_2_-95% air.

### Plasmid

The Ad plasmid for rAdF35-E1-CMV-GFP, pAdHM34-hAIB-miR142T-miR142T-EGFP-CMV, was constructed as follows. First, a human telomerase reverse transcriptase (hTERT) promoter-driven E1 gene expression cassette, in which the E1A and E1B genes were linked by an internal ribosome entry site (IRES), in pHM5-hAIB-142-3pT [6] and a cytomegalovirus (CMV) promoter-driven GFP expression cassette containing the four copies of miR-142-3p-targeted sequences were inserted into pHM5 [21], producing pHM5-hAIB-miR142T-miR142T-EGFP-CMV. The hTERT promoter-driven E1 gene expression cassette and the CMV promoter-driven GFP expression cassette were inserted into pAdHM34 [22] to obtain pAdHM34-hAIB-miR142T-miR142T-EGFP-CMV.

The Ad plasmid for rAdF35-E1-2A-GFP, pAdHM34-hAGB-miR142T, was constructed as follows. The hTERT promoter-driven E1 and GFP gene expression cassette in which the P2A peptide-coding sequence and the GFP gene were located downstream of the E1A gene was chemically synthesized (GENEWIZ Japan Corp, Tokyo, Japan) and was inserted into pAdHM34 to obtain pAdHM34-hAGB-miR142T.

The Ad plasmid of rAdF35-pIX-2A-GFP, pAdHM34-hAIB-miR142T-pIX-P2A-EGFP-miR142T, was constructed as follows. The pIX gene fused with the 2A and GFP genes and four copies of miR-142-3p-targeted sequences was chemically synthesized (GENEWIZ Japan Corp) and was inserted into pAdHM34 by homologous recombination, producing pAdHM34-pIX-2A-GFP-miR142T. An hTERT promoter-driven E1 gene expression cassette was inserted into the E1 deletion region of pAdHM34-pIX-2A-GFP-miR142T, producing pAdHM34-hAIB-miR142T-pIX-P2A-EGFP-miR142T.

The Ad plasmid of rAdF35-E1-2A-GFP-ADP, pAdHM309-E1-F35-hAGB3-miR142T, was constructed as follows. pAdHM309-E1-F35 was created by substituting the fiber gene of pAdHM309-E1 [23] with that of Ad35. The hTERT promoter-driven E1 and GFP gene expression cassette in pHM5-E1-2A-GFP-miR142T was inserted into pAdHM309-E1, producing pAdHM309-E1-F35-hAGB3-miR142T. Details of the plasmid construction are available upon request.

### Production of conditionally replication-competent Ads

Recombinant Ads were produced by transfection of the *Pac*I-digested Ad plasmids into HEK293 cells using Lipofectamine2000 (Invitrogen, Carlsbad, CA). All recombinant Ads were grown in H1299 cells, purified by two rounds of cesium chloride-gradient ultracentrifugation, dialyzed, and stored at -80°C. The virus particle (VP) titers were measured using a spectrophotometric method [24], and biological titers were determined using an Adeno-X-rapid titer kit (Clontech, Mountain View, CA). The ratio of the particle-to-biological titer was between 4.3 and 15 for each Ad used in this study.

### Flow cytometry analysis of GFP expression levels in human cultured cells

Human cultured tumor cells were seeded with 2-4x10^4^ cells in a low-binding 24-well plate and incubated with the recombinant Ads at 30 and 300 virus particles (VP)/cell. After a 24-h incubation, cells were collected, washed twice with cold PBS, and incubated with trypsin for 2 minutes at 37°C. GFP expression levels in the cells were analyzed using a flow cytometer (MACS Quant Analyzer; Miltenyi Biotec, Bergisch Gladbach, Germany). Data were analyzed by FCS Multicolor Data Analysis Software (Flowjo; BD Biosciences, San Jose, CA).

### Real-time PCR analysis of Ad genome copy numbers in human cultured cells

Human tumor cells were seeded at 4x10^4^ cells/well in a low-binding 24-well plate and were incubated with the recombinant Ads at 30 and 300 VP/cell. After a 24-h incubation, total DNA containing the Ad genome was recovered from the cells using DNAzol (Molecular Research Center, Cincinnati, OH). Ad genome copy numbers were determined by quantitative PCR using THUNDERBIRD Next SYBR qPCR Mix (TOYOBO, Osaka, Japan) as previously described [24].

### Detection of CTCs in the blood samples of lung cancer patients

CTCs in the peripheral blood samples of lung cancer patients were detected as previously described with slight modifications [1]. Briefly, cells were recovered from the 3 ml blood samples of patients with non-small cell lung cancer (NSCLC). Following enrichment of CD45-negative cells, cells were incubated with 1.0x10^8^ VP of GFP-expressing conditionally replicating Ads at 37°C for 24 h. The cells were washed and stained with phycoerythrin (PE)-labeled anti-human CD45 antibody (clone HI30; BioLegend, San Diego, CA) and then observed under a fluorescence microscope. CTCs were defined as GFP+/CD45- cells. The procedures for obtaining peripheral blood from patients with lung cancer were approved by the Institutional Review Board at the Juntendo University School of Medicine (M16-0154). All patients provided written informed consent. Information about cancer stages and histological cancer types of lung cancer patients is shown in Table 1.

**Table 1:** Clinical information of lung cancer patients.

Cancer Patient	Stage	Histological type
A	IV B	Adenocarcinoma
B	IV B	Squamous cell carcinoma
C	IV B	Adenocarcinoma
D	I A3	Adenocarcinoma
E	IV A	Squamous cell carcinoma
F	IV B	Adenocarcinoma
G	IV A	Adenocarcinoma
H	IV A	Adenocarcinoma
I	IV B	Adenocarcinoma
J	III A	Adenocarcinoma
K	IV A	non-small-cell lung cancer
L	I B	Pleomorphic carcinoma
M	IV A	Squamous cell carcinoma
N	I B	Adenocarcinoma
O	III A	Adenocarcinoma
P	IV A	Squamous cell carcinoma
Q	IV A	Adenocarcinoma

### Statistical analyses

Statistical analysis was performed using Student’s *t*-test or one-way ANOVA followed by a Tukey post hoc test. Data are presented as the means ± S.D.

## Results

### Development of novel types of GFP-expressing conditionally replicating Ads for efficient detection of CTCs

**Fig 1:**
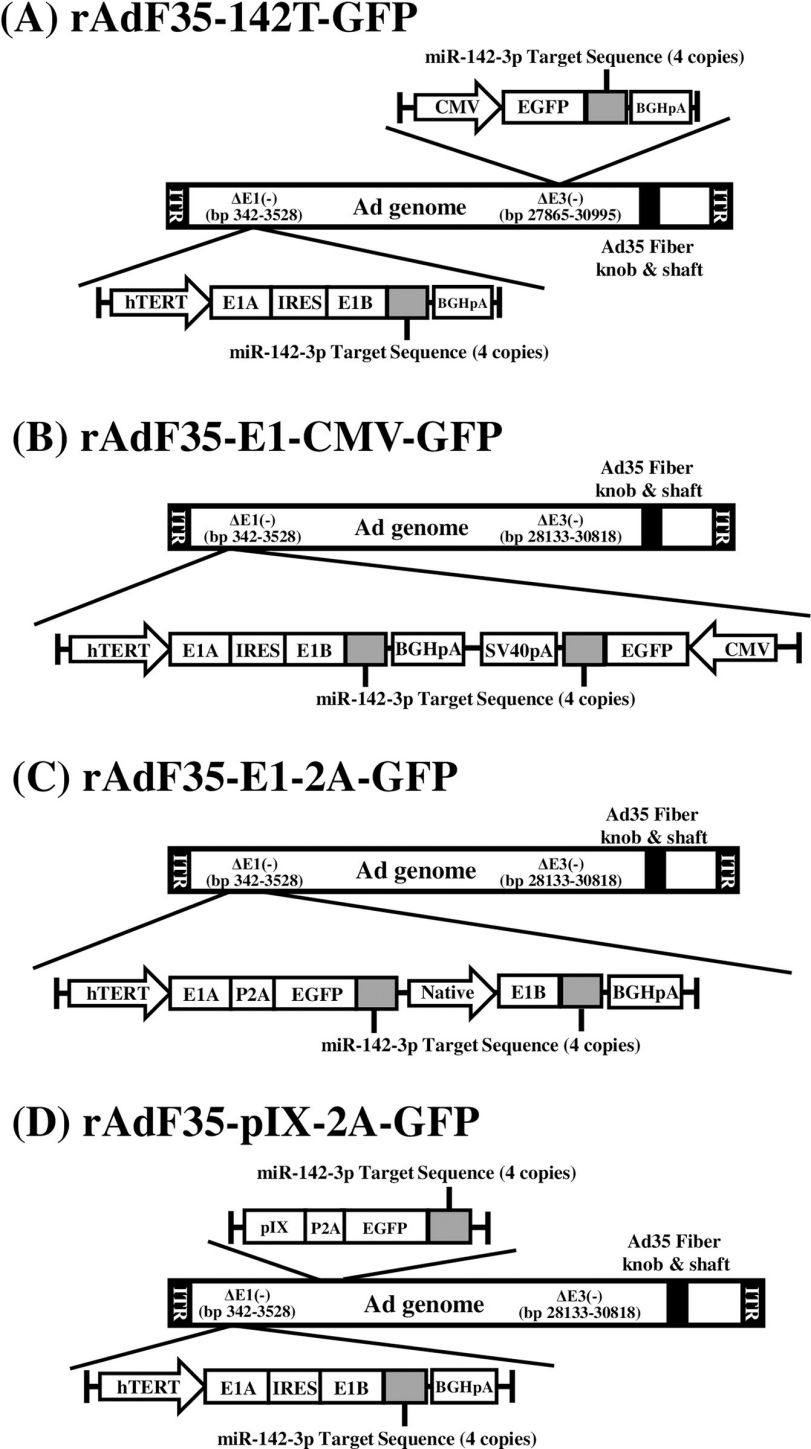
Schematic diagrams of GFP-expressing conditionally replicating Ad genomes used in this study. Ad35, adenovirus serotype 35; BGHpA, bovine growth hormone polyadenylation signal; CMV, cytomegalovirus immediate-early enhancer and promoter; EGFP, enhanced green fluorescence protein gene; hTERT, human telomerase reverse transcriptase promoter; ITR, inverted terminal repeat; IRES, internal ribosomal entry site; native, native E1B gene promoter; SV40pA, the simian virus 40 polyadenylation signal.

In order to improve the GFP expression levels in CTCs and titers of GFP-expressing conditionally replicating Ads, we first developed three types of novel conditionally replicating Ads. In the previous study, we used rAdF35-142T-GFP, which contained the CMV promoter-driven GFP expression cassette in the E3 region and miR-142-3p-targeted sequences in the 3’-UTR of the E1B gene and the GFP gene (Fig 1A) [6]. rAdF35-E1-CMV-GFP contained the CMV promoter-driven GFP expression cassette in the region downstream of the hTERT promoter-driven E1 gene expression cassette in the reverse orientation (Fig 1B). In rAdF35-E1-2A-GFP, the 2A peptide-coding sequence and the GFP gene were inserted downstream of the E1A gene in order to transcribe the GFP gene by a tumor cell-specific promoter (Fig 1C). The E1B gene was driven by the native E1B gene promoter in rAdF35-E1-2A-GFP. rAdF35-pIX-2A-GFP had the 2A peptide-coding sequence and the GFP gene fused with the pIX gene (Fig 1D). Our group previously reported that the pIX gene was efficiently expressed 24 h after infection [25]. These four types of conditionally replicating Ads, including rAdF35-142T-GFP, contained the Ad35 fiber proteins, which recognize CD46 as an infection receptor [14], and 4 copies of sequences perfectly complementary to miR-142-3p, which is expressed in a blood cell-specific manner [6, 20].

### GFP expression levels in human cultured tumor cell lines following treatment with GFP-expressing conditionally replicating Ads

**Fig 2:**
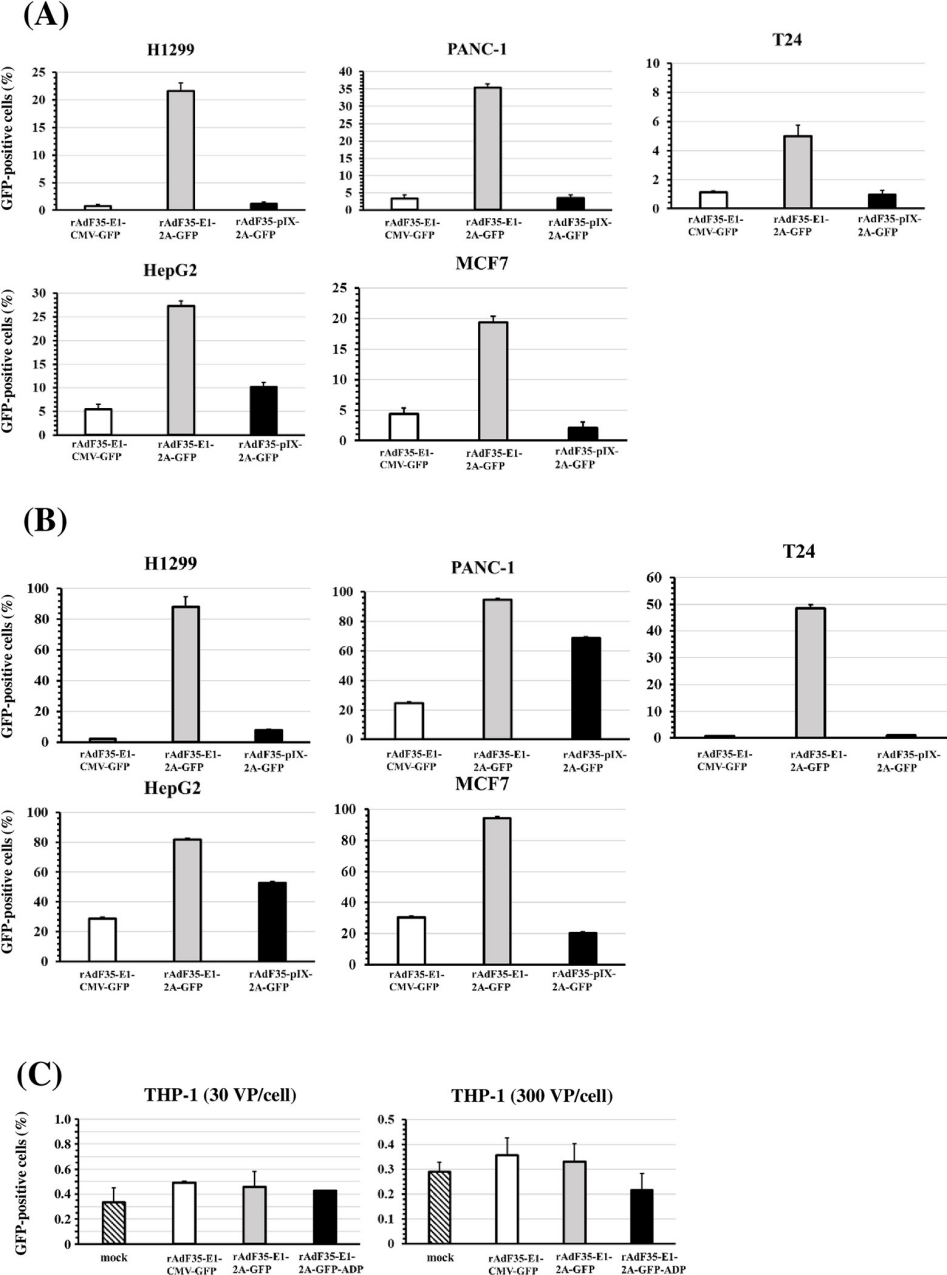
Quantitative assessment of GFP labeling efficiencies of GFP-expressing conditionally replicating Ads in human tumor cell lines and blood cells. (A, B) Percentages of GFP-positive human tumor cells following infection with GFP-expressing conditionally replicating Ads. Human tumor cell lines were infected with conditionally replicating Ads at 30 (A) and 300 VP/cell (B). After a 24-h incubation, these cells were subjected to flowcytometric analysis. Percentages of GFP-positive cells in mock-infected group were less than 1.1%. Date are expressed as the means ± S.D. (n = 3). (C) Percentages of GFP-positive human blood cells following infection with GFP-expressing conditionally replicating Ads. THP-1 cells were infected with conditionally replicating Ads at 30 and 300 VP/cell. After a 24-h incubation, these cells were subjected to flowcytometric analysis. Date are expressed as the means ± S.D. (n = 3).

In order to examine the GFP expression levels following treatment with conditionally replicating Ads, rAdF35-E1-CMV-GFP, rAdF35-E1-2A-GFP, and rAdF35-pIX-2A-GFP were added to five types of human tumor cells cultured in suspension. rAdF35-E1-2A-GFP treatment yielded the highest numbers of GFP-positive cells in all the human tumor cell lines (Fig 2A, 2B and S1 Fig). The percentages of GFP-positive tumor cells following infection with rAdF35-E1-2A-GFP at 300 VP/cell were more than 80% in all the human tumor cell lines, with the exception of T24 cells.

Next, in order to examine the GFP expression levels in blood cells following treatment with conditionally replicating Ads, cells of THP-1, a human monocyte cell line, were incubated with the recombinant Ads. Statistically significant numbers of GFP-positive cells were not found following treatment with rAdF35-E1-CMV-GFP, rAdF35-E1-2A-GFP, or rAdF35-pIX-2A-GFP (Fig 2C). These results suggested that none of the three conditionally replicating Ads produced GFP-positive blood cells. Collectively, the above results indicated that rAdF35-E1-2A-GFP realized the most efficient mediation of GFP expression in human tumor cells among the three types of conditionally replicating Ads, without detectable levels of GFP expression in blood cells.

### GFP expression levels by the conditionally replicating Ad possessing the ADP gene in human tumor cells

**Fig 3:**
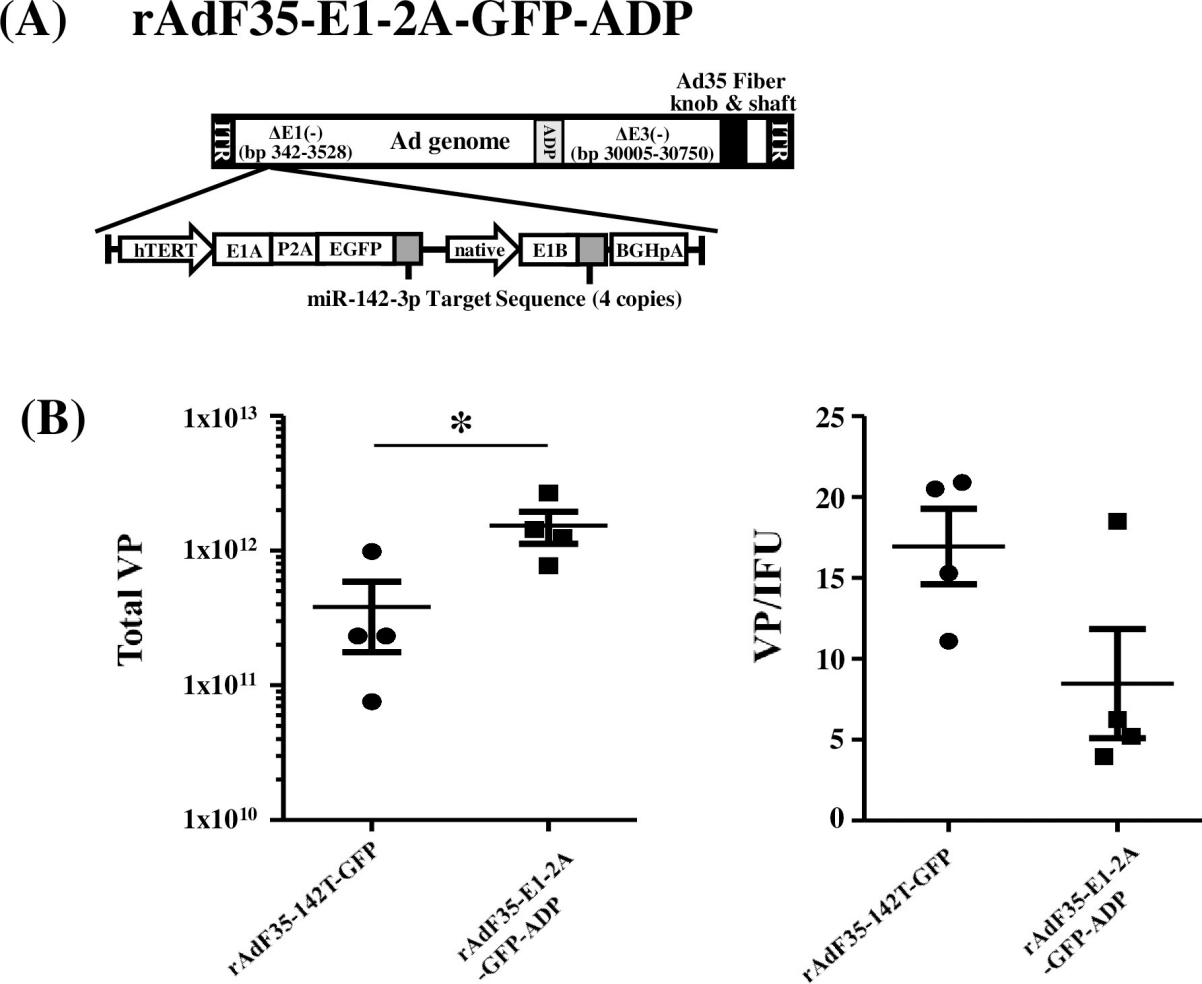
Physical and biological titers of GFP-expressing conditionally replicating Ads used in this study. GFP-expressing recombinant Ads were amplified and purified as described in Materials and Methods. (A) Total VP titers of Ads. (B) VP/IFU ratios of Ads. Date are expressed as the means ± S.D. (n = 4). *p<0.05 (Student’s *t*-test).

In order to further improve the GFP expression levels following infection with rAdF35-E1-2A-GFP in human tumor cells, the E3-deleted region in rAdF35-E1-2A-GFP was modified to leave the ADP gene in the conditionally replicating Ad genome, producing rAdF35-E1-2A-GFP-ADP (Fig 3A). The ADP gene plays a crucial role in promotion of progeny virus production [26, 27]. Titers of rAdF35-E1-2A-GFP-ADP were slightly but significantly higher (about 4-fold) than those of rAdF35-142T-GFP (Fig 3B). VP/infectious unit (IFU) titer ratios were comparable between rAdF35-E1-2A-GFP-ADP and rAdF35-142T-GFP. These data suggested that inclusion of the ADP gene in the Ad genome improved the production of conditionally replicating Ads.

**Fig 4:**
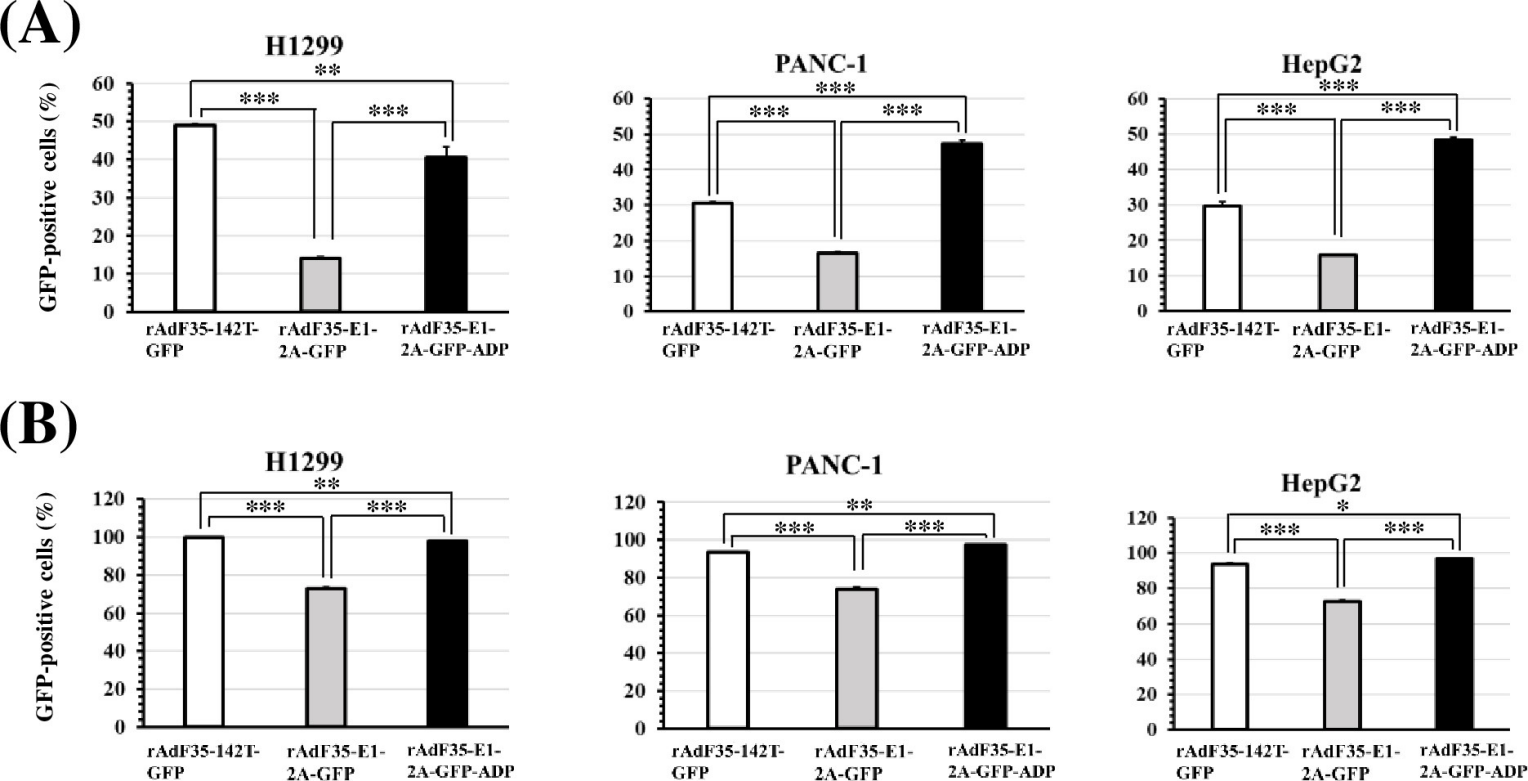
Quantitative assessment of GFP labeling efficiencies of GFP-expressing conditionally replicating Ads containing the ADP gene in human tumor cell lines. (A, B) Percentages of GFP-positive human tumor cells following infection with GFP-expressing conditionally replicating Ads containing the ADP gene. Human tumor cell lines were infected with conditionally replicating Ads at 30 (A) and 300 VP/cell (B). After a 24-h incubation, these cells were subjected to flowcytometric analysis. Percentages of GFP-positive cells in mock-infected group were less than 1.3%. Date are expressed as the means ± S.D. (n = 3). *p<0.05, **p<0.005, ***p<0.001 (one-way ANOVA followed by a Tukey post hoc test).

**Fig 5:**
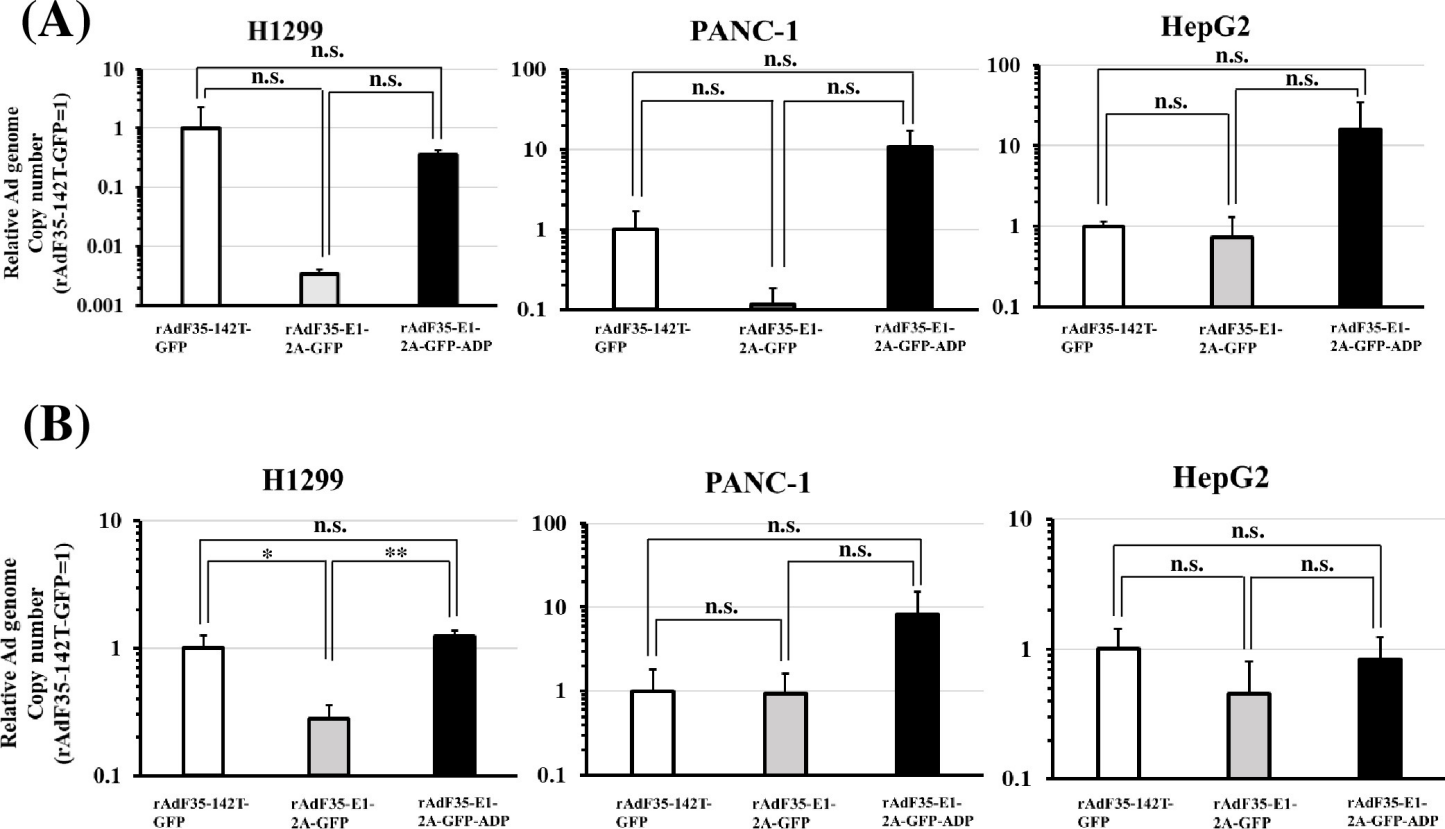
Relative Ad genome copy numbers in human tumor cells following infection with GFP-expressing conditionally replicating Ads. Human tumor cells were infected with GFP-expressing conditionally replicating Ads at 30 (A) and 300 VP/cell (B). Ad genome copy numbers following treatment with rAdF35-142T-GFP were normalized to 1. Total DNA, including the virus genome, was isolated from the cells after a 24-h incubation, followed by real-time PCR analysis. Date are expressed as the means ± S.D. (n = 3). *p<0.05, **p<0.005 (one-way ANOVA followed by a Tukey post hoc test). n.s., not significant.

**Fig 6:**
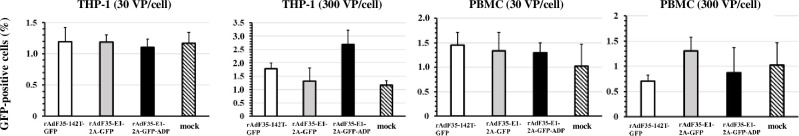
Percentages of GFP-positive human blood cells following infection with GFP-expressing conditionally replicating Ads containing the ADP gene. THP-1 cells and PBMCs were infected with conditionally replicating Ads at 30 and 300 VP/cell. After a 24-h incubation, these cells were subjected to flowcytometric analysis. Date are expressed as the means ± S.D. (n = 3).

Next, we examined the GFP expression levels in human tumor cells following treatment with rAdF35-142T-GFP, rAdF35-E1-2A-GFP, and rAdF35-E1-2A-GFP-ADP (Fig 4A, 4B and S2 Fig). rAdF35-E1-2A-GFP-ADP mediated slightly but significantly larger numbers of GFP-positive PANC-1 and HepG2 cells than the other types of the recombinant Ads, although the percentage of GFP-positive cells following infection with rAdF35-E1-2A-GFP-ADP was slightly lower than those following infection with rAdF35-142T-GFP in H1299 cells. Statistically significant differences in the virus genome copy numbers were not found in the tumor cells for the three types of the recombinant Ads, except when H1299 cells were infected at 300 VP/cell (Fig 5A and 5B). Although flowcytometric analysis showed that rAdF35-E1-2A-GFP-ADP produced significantly higher levels of GFP-positive than mock-infected THP-1 cells at 300 VP/cell (Fig 6A), GFP expression levels were below the detection limit by fluorescence microscopy (S3 Fig). In addition, detectable levels of GFP-positive PBMCs were not found following infection with the three types of the recombinant Ads. These data indicated that rAdF35-E1-2A-GFP-ADP produced comparable or slightly higher levels of GFP-positive human tumor cells, compared with rAdF35-142T-GFP, without significant levels of GFP expression in blood cells.

### Conditionally replicating Ad-mediated detection of CTCs in the blood of lung cancer patients

**Fig 7:**
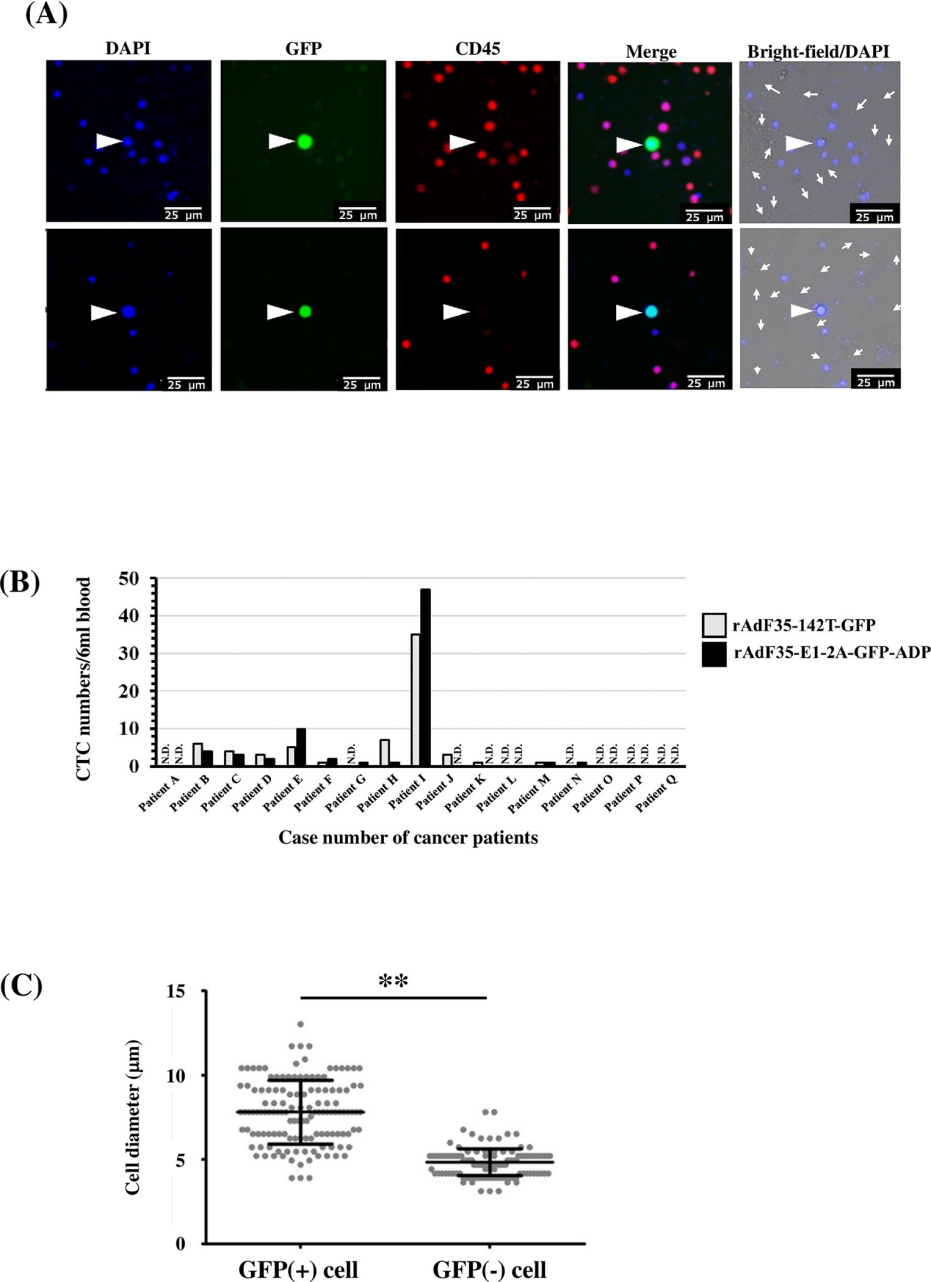
Detection of CTCs in the blood of lung cancer patients by GFP-expressing conditionally replicating Ads. Blood samples were provided from 17 lung cancer patients. Blood cells were infected with GFP-expressing conditionally replicating Ads. Blood cells were observed under a fluorescence microscope after a 24-h incubation. (A) GFP-positive cells (arrow head) in the blood of lung cancer patients following incubation with rAdF35-142T-GFP. Arrows indicate DAPI-negative cells. (B) The numbers of CTCs (GFP+/DAPI+/CD45-) in the blood samples of lung cancer patients. N.D., not detected. (C) Long diameters of CTCs (GFP+/CD45-) and blood cells (GFP-/CD45+). The cell diameters were measured under a fluorescence microscope. Data are expressed as the means ± S.D. (n = 133). **p<0.01 (Student’s *t*-test).

In order to compare the CTC detection efficiencies of rAdF35-142T-GFP and rAdF35-E1-2A-GFP-ADP in the blood of cancer patients, non-blood cells in the blood of lung cancer patients were concentrated, followed by infection with the recombinant Ads. GFP+/CD45- cells that were considered to be CTCs were detected for both types of the recombinant Ads (Fig 7A arrow head). More than 10-fold larger numbers of DAPI- cells were found, compared with DAPI+ cells (Fig 7A, arrows). The numbers of GFP+/CD45- cells following incubation with rAdF35-E1-2A-GFP-ADP and rAdF35-142T-GFP were almost comparable (Fig 7B). GFP+/CD45- cells were found in 10 of 17 patients (58.8%) for both types of recombinant Ads. No clusters of viable circulating tumor microemboli (CTM) were found in the samples in this study.

The mean longitudinal diameters of GFP+/CD45- and GFP-/CD45+ cells were 7.81 μm and 4.84 μm, respectively (Fig 7C). Previous studies reported that the diameters of CTCs were larger than those of normal blood cells [5, 28, 29]. No GFP+/CD45- cells were detected in the blood samples of healthy individuals following treatment with rAdF35-E1-2A-GFP-ADP or rAdF35-142T-GFP (S4 Fig). These results indicate that rAdF35-E1-2A-GFP-ADP and rAdF35-142T-GFP can detect CTCs at comparable efficiencies in lung cancer patients without production of false-positive cells.

## Discussion

In this study, we generated novel conditionally replicating Ads by modifying the GFP expression cassette and leaving the ADP gene in the Ad genome in order to detect CTCs more efficiently than by the recombinant Ad used for detection of CTCs in the previous study [6]. The novel recombinant Ad, rAdF35-E1-2A-GFP-ADP, that was developed in this study, and rAdF35-142T-GFP, that was developed in the previous study [6], exhibited comparable levels of CTC detection efficiencies in our present study.

In this study, 1x10^8^ VP of the recombinant Ads were applied to the non-blood cells isolated from 3 ml of blood samples after enrichment of non-blood cells, although blood cells, including leukocytes, were included. When the CTCs were observed under a florescence microscope, the total number of DAPI+ cells, including CTCs, was approximately 10^4^ cells/sample. In addition to DAPI+ cells, DAPI- cells, including platelets and erythrocytes, were also found in the samples. The numbers of DAPI- cells were significantly higher (approximately 10–50-fold) than those of DAPI+ cells (Fig 7A). All blood cells except for erythrocytes are CD46-positive. Taking these findings into consideration, approximately 10^5^ CD46-positive cells were incubated with 10^8^ VP of the conditionally replicating Ads in this study. Hence, the conditionally replicating Ads were added to the blood cells at approximately 10^3^ VP/cell. When human tumor cell lines were incubated with the conditionally replicating Ads at 300 VP/cell, more than 90% of the tumor cells were GFP-positive (Fig 4B), suggesting that sufficient amounts of the conditionally replicating Ads were added to the blood cells of cancer patients for detection of CTCs under this experimental condition.

Previous studies using rAdF35-142T-GFP, also sometimes referred to as OBP-1101, reported that CTCs were detected using rAdF35-142T-GFP in 42.5% [6] and 69.1% [1] of lung cancer patients, although the methods of treatment with the conditionally replicating Ads and observation of CTCs were slightly different between the studies. In this study, 58.8% of NSCLC patients were found to be CTC-positive by using rAdF35-142T-GFP and rAdF35-E1-2A-GFP-ADP (Fig 7B). These data indicated that the sensitivity of CTC detection in this study was comparable to those in the previous studies [1, 6]. In previous studies using a CellSearch^TM^ system, CTCs were detected in 22.5% and 43.4% of patients with NSCLC [30, 31]. Previous studies using the GFP-expressing conditionally replicating Ads containing the hTERT promoter-driven E1gene expression cassette reported that CTCs were efficiently detected in the other types of cancer patients (50.0% of obstructive colorectal cancer patients [32], 26.0% of cervical cancer patients [12], 39.6% of gynaecological cancer patients [33]), although precise experimental conditions were different between the studies. These findings suggest that a CTC detection method using a GFP-expressing conditionally replicating Ad can be utilized for detection of CTCs derived from various types of tumors.

Comparison between rAdF35-E1-2A-GFP and rAdF35-E1-2A-GFP-ADP showed that the percentages of GFP-positive tumor cells at 24 h after infection were significantly elevated by inclusion of the ADP gene (Fig 4A and 4B). The virus genome copy numbers of rAdF35-E1-2A-GFP-ADP tended to be higher than those of rAdF35-E1-2A-GFP, although statistically significant differences were not found. The ADP gene was mainly expressed at the late phase of infection and contributed to the release of progeny virus from infected cells, leading to an improvement of progeny virus infection of neighboring cells [26, 27]. On the other hand, it remained unclear whether the ADP contributed to virus genome replication. The above-described findings of our present experiments suggested that the ADP gene is involved in virus genome replication in an unknown manner and/or that progeny virus would be released from the infected cells within 24 h, followed by infection of neighboring cells. Further examination is necessary to elucidate the role of the ADP in virus genome replication. On the other hand, no apparent differences were found in the numbers of GFP-positive tumor cells and CTC detection efficiencies between rAdF35-142T-GFP and rAdF35-E1-2A-GFP-ADP (Figs 4A, 4B and 7B). Other differences observed between the Ad genomes of these two types of Ads, including the structure of the Ad genome, the promoters driving the GFP gene expression, and the presence or absence of the ADP gene affected the GFP expression levels in the tumor cells.

Recently, nanoparticle-based CTC detection systems have been developed [34–36]. Nanoparticles containing CTC-targeting ligands and antibodies on the surface efficiently captured CTCs, resulting in efficient enrichment and detection of CTCs, however, expression of CTC markers on cellular surface is essential for efficient capture of CTCs. In addition, CTC marker proteins are different between tumor cell types. Tumor cell-specific marker proteins, including hTERT, are often intracellularly expressed. Conditionally replicating Ads mediate tumor cell-specific replication *via* utilizing tumor cell-specific intracellular proteins, resulting in tumor cell-specific GFP expression.

The conditionally replicating Ads used in this study contained the hTERT promoter for the E1 gene expression. The conditionally replicating Ads efficiently replicated and killed hTERT-positive tumor cells, on the contrary, hTERT-negative normal cells were resistant to the conditionally replicating Ads [37]. hTERT activity or mRNA expression was not found in all of clinical tumor samples [38, 39]. Although it is now unclear whether hTERT was efficiently expressed in CTCs, hTERT-negative CTCs might not be efficiently detected by the conditionally replicating Ads used in this study. Conditionally replicating Ads containing another type of tumor-specific promoter should be used for detection of hTERT-negative tumor cells.

In summary, in order to detect CTCs more efficiently than by the recombinant Ad used in the previous study [6]. One of the novel recombinant Ads, rAdF35-E1-2A-GFP-ADP, exhibited CTC detection efficiency comparable to that of rAdF35-142T-GFP developed in the previous study. This study provides important clues for the future development of recombinant Ad-based systems for the evaluation of biomarkers.

## Supporting information

S1 Fig Flow cytometric analysis of GFP expression in human tumor cell lines following incubation with GFP-expressing conditionally replicating Ads at 30 VP/cell (A) and 300 P/cell (B). GFP expression levels in the human tumor cells were measured using flow cytometry after a 24-h incubation. The numbers in the graph indicate mean fluorescence intensities of GFP. The representative data of at least three measurements was shown.
(PDF)

S2 Fig Flow cytometric analysis of GFP expression in human tumor cell lines following incubation with GFP-expressing conditionally replicating Ads containing the ADP gene at 30 VP/cell (A) and 300 P/cell (B). GFP expression levels in the human tumor cells were measured using flow cytometry after a 24-h incubation. The numbers in the graph indicate mean fluorescence intensities of GFP. The representative data of at least three measurements was shown.
(PDF)

S3 Fig GFP expression in THP-1 cells of healthy volunteers following incubation with GFP-expressing conditionally replicating Ads. THP-1 cells were observed under a fluorescence microscope after a 24-h incubation. Scale bars indicate 25 μm.
(PDF)

S4 Fig GFP expression in the blood cells of healthy volunteers following incubation with rAdF35-142T-GFP and rAdF35-E1-2A-GFP-ADP. Blood cells were observed under a fluorescence microscope after a 24-h incubation. Arrow heads indicate dust-like particle. Scale bars indicate 25 μm.
(PDF)
