# Supplementary figures and images for: Novel conditionally replicating adenovirus-mediated efficient detection of circulating tumor cells in lung cancer patients
Source: PLoS One. 2023 Oct 19;18(10):e0286323. doi: 10.1371/journal.pone.0286323 (PMC10586684; doi:10.1371/journal.pone.0286323)

S Fig 1

(A)

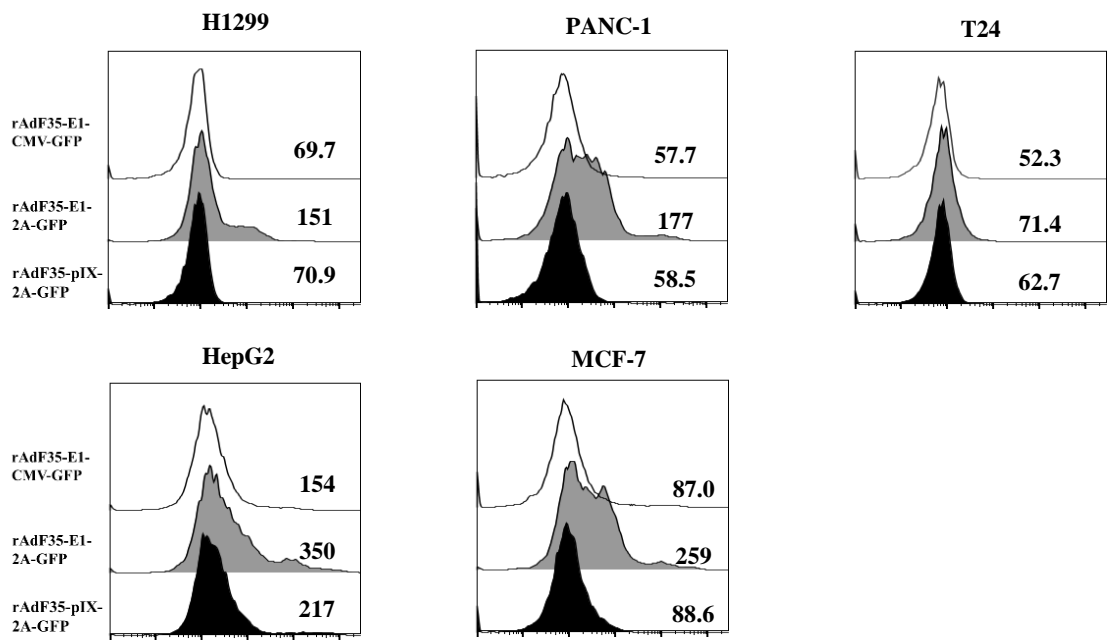

(B)

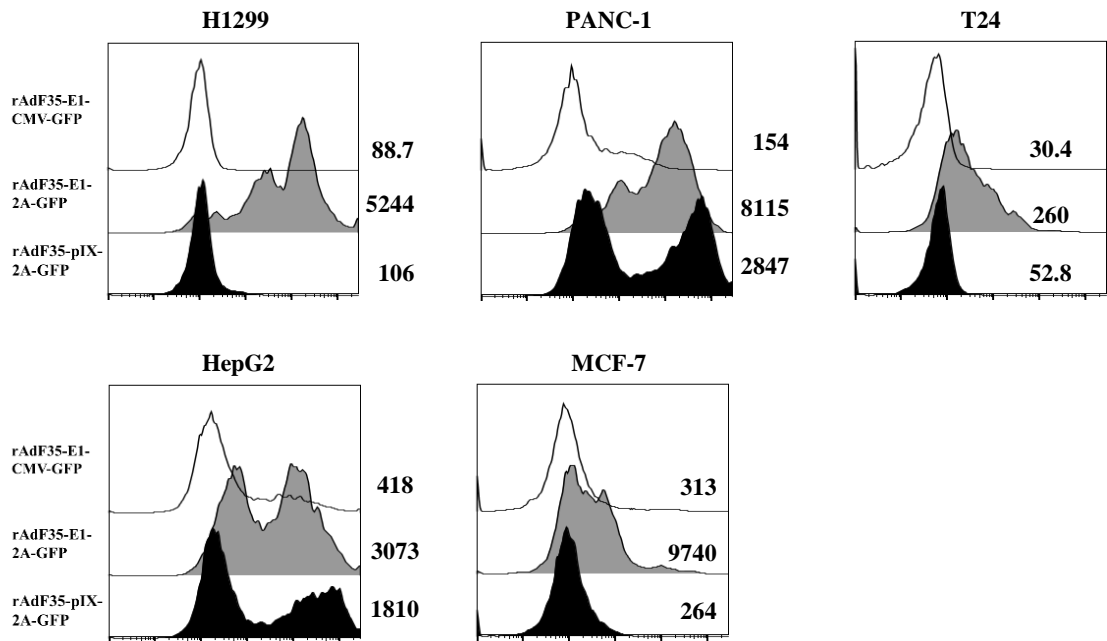

Supplement: S1 Fig — Flow cytometric analysis of GFP expression in human tumor cell lines following incubation with GFP-expressing conditionally replicating Ads at 30 VP/cell (A) and 300 P/cell (B). GFP expression levels in the human tumor cells were measured using flow cytometry after a 24-h incubation. The numbers in the graph indicate mean fluorescence intensities of GFP. The representative data of at least three measurements was shown. (PDF) [file pone.0286323.s001.pdf]

S Fig 2

(A)

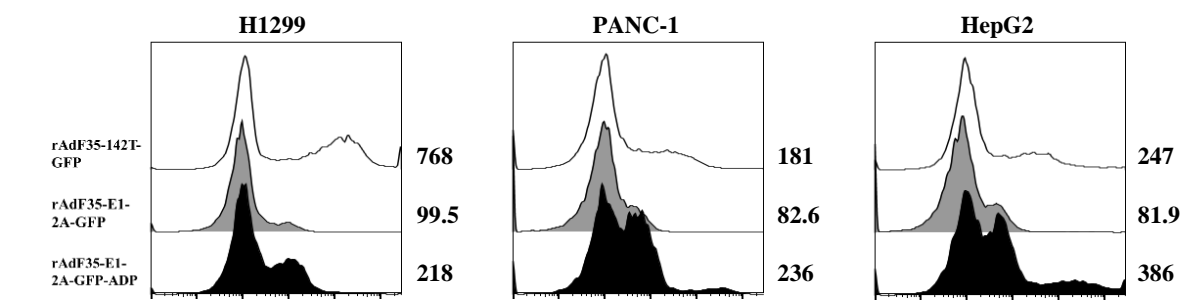

(B)

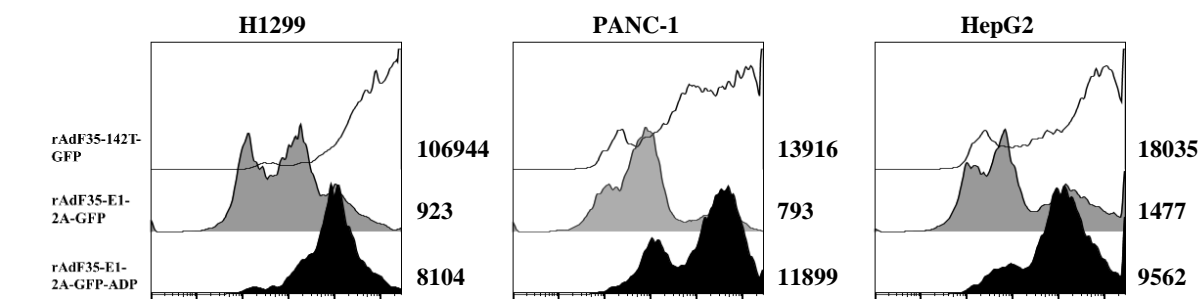

Supplement: S2 Fig — Flow cytometric analysis of GFP expression in human tumor cell lines following incubation with GFP-expressing conditionally replicating Ads containing the ADP gene at 30 VP/cell (A) and 300 P/cell (B). GFP expression levels in the human tumor cells were measured using flow cytometry after a 24-h incubation. The numbers in the graph indicate mean fluorescence intensities of GFP. The representative data of at least three measurements was shown. (PDF) [file pone.0286323.s002.pdf]

**S Fig 3**

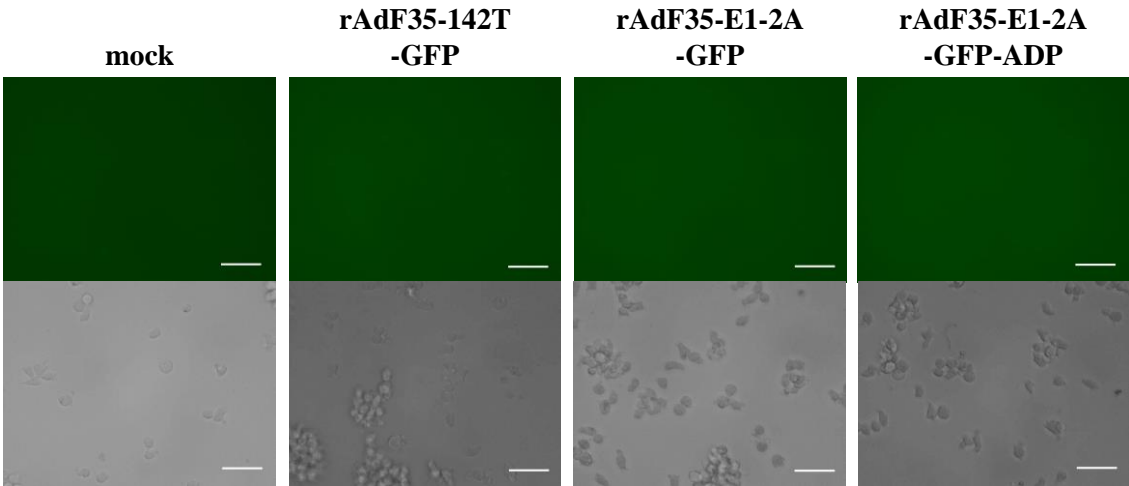

Supplement: S3 Fig — THP-1 cells were observed under a fluorescence microscope after a 24-h incubation. Scale bars indicate 25 μm. (PDF) [file pone.0286323.s003.pdf]

**S Fig 4**

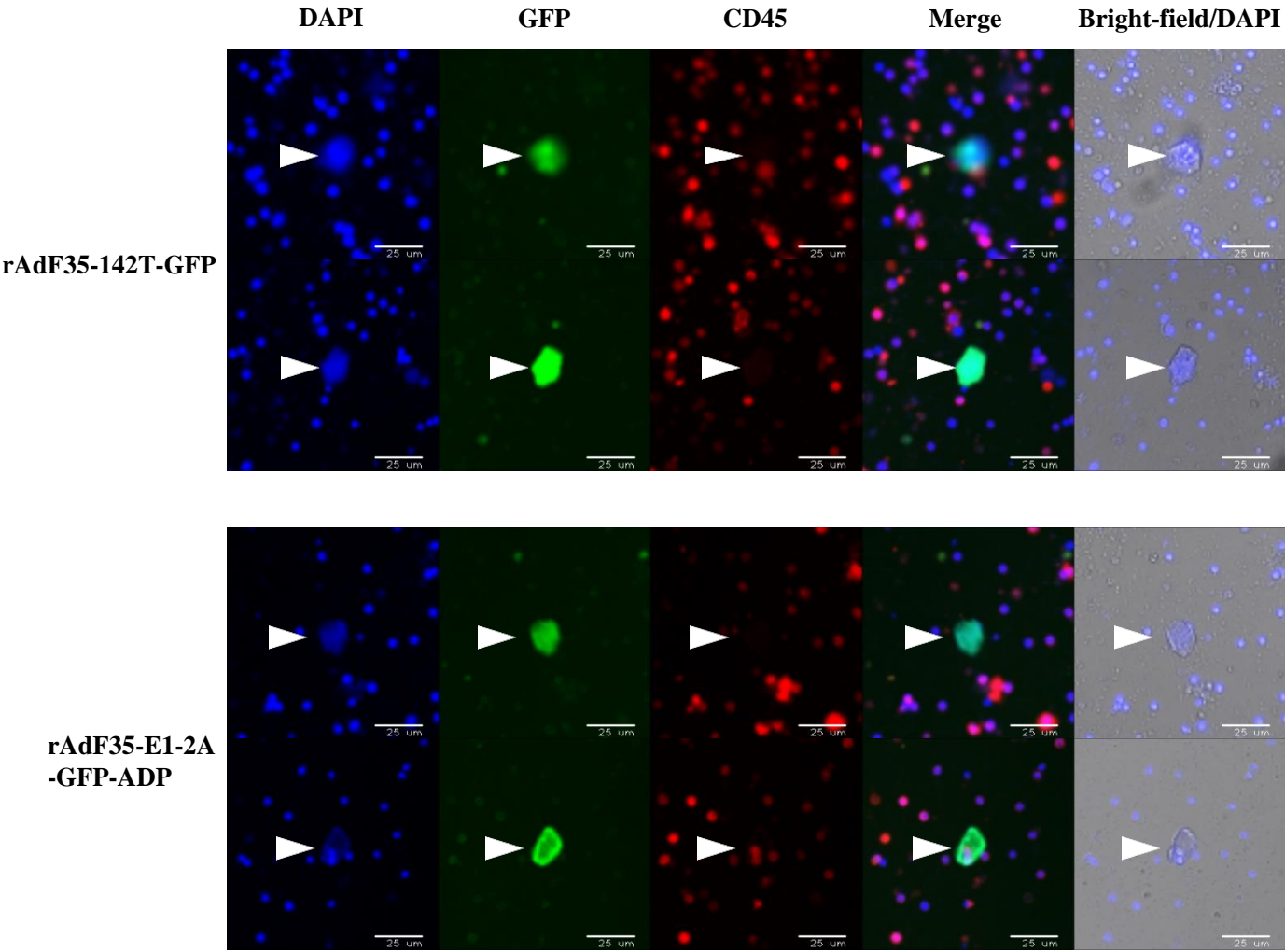

Supplement: S4 Fig — Blood cells were observed under a fluorescence microscope after a 24-h incubation. Arrow heads indicate dust-like particle. Scale bars indicate 25 μm. (PDF) [file pone.0286323.s004.pdf]
